# Derivation of a Freshwater Quality Benchmark and an Ecological Risk Assessment of Ferric Iron in China

**DOI:** 10.3390/toxics13060475

**Published:** 2025-06-04

**Authors:** Qijie Geng, Fei Guo

**Affiliations:** 1 College of Natural Resources and Environment, Northwest A&F University, Xianyang 712100, China; 2 State Key Laboratory of Environmental Criteria and Risk Assessment, Chinese Research Academy of Environmental Sciences, Beijing 100012, China

**Keywords:** ferric iron (Fe^3+^), water quality criteria/standard (WQC/WQS), ecological risk assessment (ERA), species sensitivity distribution (SSD) method

## Abstract

Acid drainage resulting from mining operations has led to significant iron contamination in surface waters, posing serious ecological and public health hazards. Elevated iron levels in freshwater ecosystems can severely affect aquatic organisms and human health. However, there remains a considerable gap in the establishment of benchmark values and ecological risk assessments (ERAs) for iron in surface waters in China. This study collected and screened 47 acute and chronic toxicity data points of 22 species for ferric iron (Fe^3+^) from various studies and databases. Three widely utilized methodologies were applied to derive long-term and short-term water quality criteria (LWQC and SWQC, respectively) for Fe^3+^; the logistic fitting curve based on the species sensitivity distribution (SSD) method was identified as the most optimal method, yielding an acute *HC*_5_ of 689 μg/L and an SWQC of 345 μg/L. The LWQC of Fe^3+^ was estimated to be 28 μg/L by dividing *HC*_5_ by the acute-to-chronic ratio (*ACR*), owing to the inadequacy of chronic toxicity data for model fitting. Utilizing these benchmarks, an ecological risk assessment (ERA) was conducted to compare the benchmarks with 68 iron exposure data points collected from surface waters across 30 provinces from eight river basins of China. The findings of 30% of the acute risk quotients and 83% of the chronic risk quotients raise substantial ecological concerns, primarily regarding the Yellow River Basin, Huaihe River Basin, and Songhua and Liaohe River Basin. This research provides critical insights into Fe^3+^ toxicity data collection and benchmark derivations, offering a benchmark data foundation for the remediation of surface water iron contamination and water quality management in China.

## 1. Introduction

Iron (Fe) is an extensively used heavy metal element, and it is the second most abundant metallic element in the Earth’s crust [1]. China has abundant iron mineral resources [2], and iron contamination is common. In correlated studies, excessive iron concentrations were found in the river body of the Middle Yangtze River [3], and the annual maximum iron concentration was monitored up to 2.38 mg/L in the man-made Changtan Reservoir in Zhejiang Province, far exceeding the standard limits in China [4]. Studies on anthropogenic enclosed agriculture wetlands in eastern Liaoning Province declared that the extension of land reclamation, continuous agricultural fertilization, and industrial activities, such as papermaking, urban expansion, etc., greatly increased the accumulation of heavy metals [5]. Since the 1950s, population growth, economic development, extended mineral exploitation, large-scale water conservancy projects, etc., have led to more terrestrial-generated materials entering surface water bodies, which has caused a sharp increase in freshwater iron concentration in China [3]. In order to control and remediate iron contamination effectively, the Chinese government set the criteria for iron concentration in surface water systems to 0.3 mg/L in the Environmental quality standard for surface water (GB3838-2002) and Standard for drinking water quality (GB5749-2022) [6,7].

In ecology, iron plays an indispensable role in all organisms’ growth and maintenance functions, and it has been found to rank first in both significance and amount among 14 trace elements that humans need [1]. However, both monomeric and chemosynthetic iron are toxic or weakly toxic [8], indicating that excessive iron concentrations in the environment cause toxic effects to organisms and human health. Related studies have proven that dissolved or precipitated iron in surface water, once ingested by organisms, is hard to metabolize, decompose, or expel from the body, and it can easily accumulate in aquatic animals’ livers and kidneys [9] or cause sensitive reactions and persistent in-body storage in zoobenthos like shellfish [10,11], as well as bio-magnify along the food chain [12].

Ecological risk assessment (ERA) is a process of evaluating the likelihood that adverse ecological effects will occur or are occurring under exposure to stressors. It aids in anticipating future effects on both ecosystems and human health [13]. ERA has been efficiently applied in water quality measurement, prioritizing management actions, water quality standard amendment, and improvement tracking [14]. In recent years, relevant studies in China have conducted risk assessments of heavy metal concentrations in surface waters. For instance, Zhao et al. investigated metal elements in the Fenhe River, Shanxi Province, and conducted health risk assessments on adults and minors [15]. However, relevant benchmarks and thresholds of iron concentrations related to aquatic organisms remain largely unknown, especially compared to the understanding of other well-known heavy metals and metalloids, such as lead (Pb), mercury (Hg), cadmium (Cd) [16], and manganese (Mn) [17]. Moreover, redox-sensitive metals could change their speciation through valence transitions and state transitions or through interactions with other materials to realize their geochemical cycle [8]. Based on this, in North America, William A. Stubblefield, A. S. Cardwell, and Robert W. Gensemer investigated the acute and chronic toxicity of aluminum (Al) [18,19] and iron [20] to aquatic organisms. They established international water quality criteria (WQC) through the species sensitivity distribution (SSD) approach and considered different conditions (pH, hardness, dissolved organic carbon, etc.) [21]. In comparison to international academic frontiers, there are still gaps in Chinese research about metallic WQC in terms of comprehensiveness and applicability.

In natural water bodies, iron primarily exists in two oxidation states: ferrous iron (Fe^2+^) and ferric iron (Fe^3+^). Fe^3+^ has greater stability in aqueous environments characterized by severe oxidizing conditions, while Fe^2+^ is more abundant in reducing settings [22]. In surface water systems, especially in oxygenated waters, Fe^3+^ is the predominant form. For instance, in acidic mine drainage (AMD)-contaminated rivers, Fe^3+^ concentrations were found to be significantly elevated, frequently precipitating as hydroxides or iron oxides [23]. Additionally, Fe^3+^ demonstrates significant bioavailability in aquatic ecosystems and acts as an essential supply of iron for metabolic functions in various organisms. Currently, various studies have examined the ecotoxicity of ferric iron in surface water ecosystems; nevertheless, the systematic completion of data collection remains inadequate. Numerous studies have focused on regions or categories of aquatic environments, which have been extremely limited. In several countries and locations, water quality criteria often rely on total iron concentrations without distinguishing between valence states. Similarly to the research to on Sb^3+^ [24], investigating the toxicological impacts of Fe^3+^ on diverse aquatic species is necessary.

This study aimed to conduct ERA on ferric iron (Fe^3+^)’s toxic effects in surface water around China. In this study, acute and chronic toxicity data of ferric iron were collected from databases, including the Ecotoxicology Database (ECOTOX), the China National Knowledge Infrastructure, and Web of Science (WOS), to obtain aquatic biological benchmarks using three derivation methods commonly used internationally. The aquatic biological benchmarks were used to compare the collected exposure concentrations of iron in surface water bodies and centralized drinking water sources around China and to assess the potential ecological risks and human health risks of the iron concentration in China. Moreover, because the referenced Fe toxicity data were on dissolved Fe or filterable Fe, where other forms of Fe such as colloidal FeOOH were included, the derived LWQC and SWQC were higher, affecting the correlated risk quotient results. This study provides a data basis for the formulation of iron benchmarks and the revision of standards in China, supporting risk assessment and improving regional water quality management and aquatic organism protection.

## 2. Materials and Methods

### 2.1. Toxicity Data and Fe Exposure Data

#### 2.1.1. Toxicity Data

The data on the toxicity of ferric iron to aquatic organisms in this study were obtained from the Ecotoxicology Database (ECOTOX) [25], the China National Knowledge Infrastructure, and Web of Science (WOS). Based on guidelines for evaluating ecotoxicity data, the search keywords were set as follows: “iron (Fe)” and “toxicity” or “ecotoxicity”. Acute toxicity endpoints were selected as 50% of effective concentration (EC50), 50% of inhibitory concentration (IC50), and 50% of lethal concentration (LC50), while chronic toxicity endpoints were selected as No observed effect concentration (NOEC), Lowest observed effect concentration (LOEC), and Maximum acceptable toxicant concentration (MATC) [26]. The following guidelines were also considered: (1) The species is genetically stable, with great sensitivity and consistency of toxic reactions, and can be domesticated in the laboratory. (2) Requisite information is needed, including the Latin name of the species, the source, the exposure conditions, the test operating procedures, endpoints, etc. (3) Preferential selection was made for data from dependable statistical analyses.

The toxicity experimental data of ferric iron were collected and screened. Toxicity data of eligible species were screened according to kingdom, phylum, family, genus, and species. Species of different categories were screened according to exposure duration difference, and the specific screening principles referred to [27].

Eventually, toxicity data of ferric iron to freshwater aquatic organisms that met the requirements were obtained. For the same species, multiple toxicity data points with the same toxicity endpoints and the geometric mean of all the endpoints, except the eliminated outliers, were calculated as the species’ toxicity value [28]. For the same genus, the geometric mean value of all the species’ toxicity values in the genus is the genus’s toxicity value.

#### 2.1.2. Exposure Data

Surface water exposure data of ferric iron were obtained from research articles and academic dissertations published between 2009 and 2024 in WOS (CNKI, Wanfang Data Base, etc.). Given that the vast majority of dissolved iron behaves like ferric iron in surface water, we collected the exposure data of total iron to represent this. The data cover a range of surface water bodies in 30 of China’s 34 provinces (except Taiwan Province, the Hong Kong Special Administrative Region, the Macao Special Administrative Region, and Inner Mongolia), taken from 49 articles. Surface water bodies of rivers, streams, lakes, reservoirs, estuaries, and centralized drinking water sources were included. The following points were taken into account when selecting data to facilitate data reliability and relevance: (1) Data regarding the mean, standard deviation (SD), maximum value, minimum value, etc., were selected preferentially [29]. (2) Data from surface water bodies close to a contaminated site and/or an emergency event were eliminated. (3) From the same location, Fe exposure data of different river beaches or different precipitation seasons were separated.

### 2.2. Derivation Methods for the Water Quality Criteria

Aquatic life criteria cover the maximum permissible concentration at which contamination would not cause long-term or short-term adverse effects to aquatic organisms and the environmental service of the surface water. This concentration level is one of the core components of WQC [30]. Three methods are commonly used in the world to derive aquatic life criteria: the assessment factor (AF) method, the toxicity percentage ranking (TPR) method, and the species sensitivity distribution (SSD) method [31].

#### 2.2.1. The Assessment Factor (AF) Method

Among the three methods, the AF method was developed the earliest and is used to derive the aquatic life criteria. It is based on the long-term experience of the risk assessment of chemical substances. The toxicity data of the most sensitive organism are multiplied by the corresponding assessment factor or substituted into the corresponding empirical formula to obtain a single value [32]. This method is used to determine the concentration threshold of pollutants that cannot be exceeded under any circumstances, which relies on the toxicity data of the most sensitive organism [33]. Requiring less toxicity data and a simple calculation process, the AF method has strong universality when toxicity data are insufficient [34].

#### 2.2.2. The Toxicity Percentage Ranking (TPR) Method

The toxicity percentage ranking (TPR) method was gradually established with the understanding of the physical and chemical characteristics of pollutants and the development of ecotoxicology and derived by the US EPA in 1985 [30]. The method takes into account acute toxic effects (ATEs) and chronic toxic effects (CTEs). The criteria values derived include the criteria maximum concentration (CMC) and criteria continuous concentration (CCC). CMC relates to the ATEs of pollutants on aquatic animals, and CMC is 1/2 of the final acute toxic value (FAV, mg/L). CCC considers the CTEs of pollutants on aquatic animals, living plants, and bioaccumulation effects. It takes the minimum value of the final chronic toxic value (FCV, mg/L), final plant value (FPV, mg/L), and final residual value (FRV, mg/L) [35].

In accordance with the requirements of the TPR method, the species mean acute value (SMAV), genus mean acute value (GMAV), and cumulative probability (*p*) of each species are calculated, where the SMAV and GMAV are calculated using the geometric mean method [36]. The GMAVs are ranked in ascending order. Four genera with cumulative probabilities (*p*) close to 0.5 were selected for the CMC and CCC calculation:

(1) $$\begin{array}{c} \mathrm{CMC}=\frac{{HC}_{5}}{AF} \end{array}$$

CMC—criteria maximum concentration, mg/L.

*HC*_5_—hazardous concentration for 5% of the species, mg/L.

*AF*—assessment factors, 2.

#### 2.2.3. The Species Sensitivity Distribution Method

The species sensitivity distribution (SSD) method was proposed by the US EPA in 1978 for the derivation of aquatic life criteria and has been widely used in ERA [37]. Different from models of a single species combined with a single pollutant, the SSD method reflects the dose–response relationship of contamination and species by fitting an SSD curve, calculating *HC*_5_ (the concentration of pollutants harmful to 5% of the species), and constructing an SSD model [38]. Therefore, the SSD method analyzes the harm degree of different contaminations to different species from the perspective of the ecosystem, contributing to the evaluation of ecological risks.

According to software recommended by Ministry of Ecology and Environment of the People’s Republic of China: National Ecological and Environmental Benchmark Calculation Software—Species Sensitivity Distribution Method (1.0), the endpoints of the acute toxicity data obtained for this study include LC50, IC50, and EC50; the obtained chronic toxicity data include NOEC, LOEC, MATC, EC50, and LC50. The geometric mean of the acute or chronic endpoints for each species was found to be *AVE* and *CVE*, respectively. The logarithms, *lgAVE* and *lgCVE*, were calculated and ranked from minimum to maximum. The rank order R was determined, and the cumulative frequency *F_R_* of each species was calculated according to the formula below:

(2) $$\begin{array}{c} F_{R}=\frac{\sum_{1}^{R}f}{\sum f+1}\times 100\% \end{array}$$

*F_R_*—cumulative frequency, %.

*R*—rank of toxicity value, dimensionless.

*f*—frequency, the number of species corresponding to the rank R of the toxicity value.

A fitted SSD model (normal distribution model, lognormal distribution model, logistic Steele distribution model, logistic Steele distribution model, etc.) was developed using lgAVE or lgCVE as the independent variable “x” and the corresponding cumulative frequency *F_R_* as dependent variable “y”. Based on the coefficient of determination (R^2^), the root mean square (RMSE), and the probability *p*-value of the model fit (A–D test), the optimal fitting model was determined.

In the best-fitted SSD model, the lgAVE and lgCVE corresponding to the cumulative frequency of 5% were determined, the *AVE* and *CVE* were obtained through antilogarithm, *SHC*_5_ and *LHC*_5_ were obtained, and the short-time water quality criteria (SWQC) and long-time water quality (LWQC) were calculated as follows:

(3) $$\begin{array}{c} SWQC=\frac{{\mathrm{SHC}}_{5}}{\mathrm{SAF}} \end{array}$$

where SWQC denotes the short-term water quality criteria, measured in μg/L or mg/L; *SHC*_5_ denotes the 5% species hazard concentration based on acute toxicity data, in μg/L or mg/L; and *SAF* is the short-term assessment factor, which is dimensionless.

(4) $$\begin{array}{c} LWQC=\frac{{\mathrm{LHC}}_{5}}{\mathrm{LAF}} \end{array}$$

where LWQC denotes the long-term water quality criteria, in μg/L or mg/L; *LHC*_5_ denotes the 5% species hazard concentration based on chronic toxicity data, in μg/L or mg/L; and *LAF* is the long-term assessment factor, which is dimensionless.

The short-term criteria assessment factor (*SAF*) and long-term baseline assessment factors (*LAFs*) were determined based on the number of endpoints and the range of organisms covered, as well as the distribution of the fitted data.

The steps of the three criteria derivation methods are approximately equivalent in data collection and derivation calculation. However, there are differences among the three methods in the treatment of toxicity data and calculation formulas. In order to obtain the most appropriate criteria value, it is necessary to compare the results of the three methods comprehensively.

#### 2.2.4. The Risk Quotient Method

The risk quotient method is a simple method widely used to characterize risk by comparing measured or predicted exposure toxicity data to those of a certain pollutant with the predicted no-effect criteria to obtain a quotient value [39]. Risk is assessed according to the quotient. The higher the *Q_R_*, the higher the risk, which is generally considered to be significantly affected if *Q_R_* > 1 [40]. The formula is as follows:

(5) $$\begin{array}{c} Q_{R}=\frac{\mathrm{MEC}}{\mathrm{PNEC}} \end{array}$$

where *MEC* is the measured environmental concentration, measured in μg/L; PNEC is the recommended interim predicted no-effect concentration, equal to WQC, measured in μg/L.

## 3. Results and Discussion

### 3.1. Toxicity Data Collection and Screening

In this study, 1352 toxicity data points of ferric iron were screened from the Ecotoxicology Database (ECOTOX), among which 147 toxicity data points of ferric iron and 47 toxicity data points of 22 species were obtained after further screening, as shown in Table 1. The acute toxicity data of ferric iron involved 18 species in 17 genera, with a total of 31 data points, which mainly included *Fleabane trevally*, *Reticulated trevally*, *Positive trematode*, *Blackmouth Softhead minnow*, and *Bluegill sun perch*. The toxicity data values ranged from 123 μg/L to 1 × 10^6^ μg/L. Chronic toxicity data for ferric iron involved four species from four genera, with a total of 16 toxicity data points, including *Chlorella*, *Trematoda*, *Coelenterata*, and *Minnow*, whose range was distributed between 10 μg/L and 6 × 10^3^ μg/L.

The acute toxicity data were sufficient and covered a wide range of species types, allowing the data to be directly used to derive the long-term water quality criteria (LWQC) for ferric iron, but the chronic toxicity data were insufficient to directly derive the short-term water quality criteria (SWQC) for ferric iron [41], requiring calculation based on the LQWC and acute-to-chronic ratios (*ACRs*).

**Table 1:** Data of ferric iron toxicity to aquatic organisms.

Category	Latin Name	Genus	Endpoint	Toxicity Data of the Species (μg/L)	Toxicity Data of the Genera (μg/L)	Reference
Acute Toxicity	*Asellus aquaticus*	*Asellus*	EC50	124,000	124,000	[42]
	*Ceriodaphnia dubia*	*Ceriodaphnia*	LC50	36,700	33,200	[43]
	*Ceriodaphnia dubia*	*Ceriodaphnia*	LC50	30,100		[43]
	*Chironomus javanus*	*Chironomus*	LC50	1650	1650	[44]
	*Daphnia magna*	*Daphnia*	LC50	76,000	15,600	[45]
	*Daphnia magna*	*Daphnia*	EC50	9600		[46]
	*Daphnia magna*	*Daphnia*	LC50	21,000		[47]
	*Daphnia pulex*	*Daphnia*	LC50	12,900		[48]
	*Daphnia pulex*	*Daphnia*	LC50	15,800		[48]
	*Daphnia pulex*	*Daphnia*	LC50	17,400		[48]
	*Daphnia pulex*	*Daphnia*	LC50	9000		[49]
	*Daphnia pulex*	*Daphnia*	LC50	2800		[49]
	*Duttaphrynus melanostictus*	*Duttaphrynus*	LC50	400	400	[50]
	*Gambusia affinis*	*Gambusia*	LC50	133,000	99,200	[51]
	*Gambusia affinis*	*Gambusia*	LC50	74,000		[51]
	*Lepomis macrochirus*	*Lepomis*	LC50	20,300	20,300	[48]
	*Melanoides tuberculata*	*Melanoides*	LC50	8490	8490	[50]
	*Nais elinguis*	*Nais*	LC50	123	123	[50]
	*Orconectes limosus*	*Orconectes*	LC50	32,000	32,000	[52]
	*Physa gyrina*	*Physa*	LC50	12,100	12,100	[48]
	*Pimephales promelas*	*Pimephales*	LC50	21,800	21,800	[48]
	*Ptychocheilus oregonensis*	*Ptychocheilus*	LC50	54,800	54,800	[48]
	*Salmo trutta*	*Salmo*	LC50	28,000	36,300	[53]
	*Salmo trutta*	*Salmo*	LC50	47,000		[53]
	*Stenocypris major*	*Stenocypris*	LC50	279	279	[50]
	*Tubifex tubifex*	*Tubifex*	EC50	102,000	94,300	[54]
	*Tubifex tubifex*	*Tubifex*	EC50	86,100		[55]
	*Tubifex tubifex*	*Tubifex*	EC50	95,400		[55]
	*Tubifex tubifex*	*Tubifex*	EC50	71,300		[55]
	*Tubifex tubifex*	*Tubifex*	EC50	125,000		[55]
	*Xenopus laevis*	*Xenopus*	EC50	1,000,000	1,000,000	[56]
Chronic Toxicity	*Chlorella vulgaris*	*Chlorella*	LOEC	6000	4240	[57]
	*Chlorella vulgaris*	*Chlorella*	NOEC	3000		[57]
	*Daphnia pulex*	*Daphnia*	LOEC	1310	958	[48]
	*Daphnia pulex*	*Daphnia*	LOEC	1310		[48]
	*Daphnia pulex*	*Daphnia*	LOEC	1310		[48]
	*Daphnia pulex*	*Daphnia*	MATC	960		[48]
	*Daphnia pulex*	*Daphnia*	MATC	960		[48]
	*Daphnia pulex*	*Daphnia*	MATC	960		[48]
	*Daphnia pulex*	*Daphnia*	NOEC	700		[48]
	*Daphnia pulex*	*Daphnia*	NOEC	700		[48]
	*Daphnia pulex*	*Daphnia*	NOEC	700		[48]
	*Lecane quadridentata*	*Lecane*	LOEC	100	31.6	[58]
	*Lecane quadridentata*	*Lecane*	NOEC	10		[58]
	*Pimephales promelas*	*Pimephales*	LOEC	1010	569	[48]
	*Pimephales promelas*	*Pimephales*	MATC	570		[48]
	*Pimephales promelas*	*Pimephales*	NOEC	320		[48]

### 3.2. WQC Derivation

#### 3.2.1. Criteria of the Assessment Factor (AF) Method

According to the derivation steps of the AF method, the acute toxicity value (*ATV*) and chronic toxicity value (*CTV*) of Fe^3+^ were calculated. From the acute toxicity data in Table 1, it can be seen that the most sensitive organism is *Nymphobranchus elegans*, from which we can derive an *ATV* of 123 μg/L. Moreover, the acute and chronic toxicity data of *Flea-flycatchers* and *the black-headed soft-mouthed minnow* were obtained under the same experimental conditions as this study. Therefore, the geometric mean of *ACR* was determined to be 24.7. Correspondingly, the WQC of Fe^3+^ was derived by dividing the *ATV* of *Nymphobranchus elegans* by the geometric mean of the *ACR*. The obtained WQC was 4.98 μg/L.

#### 3.2.2. Results of the Toxicity Percentage Ranking Method

Based on the acute toxicity data of ferric iron in Table 1, the four genera with a *p* value close to 0.05 were *Cynomolgus*, *Narrow star mesopotamus*, *Cephalopod toad*, and *Anopheles*. From the formula of the TPR method, the acute toxicity value (*ATV*) of ferric iron was 222 μg/L, and therefore, the criteria maximum concentration (CMC) of Fe^3+^ was 111 μg/L. The chronic toxicity value (*CTV*) of ferric iron was transformed using the *ACR* and calculated to be 9.0 μg/L. Since no toxicity data of plants that met the requirements were collected, this study could not obtain the final plant chronic value (FPV) of ferric iron. Moreover, since the current food safety standard in China does not indicate an Fe^3+^ concentration value, the final residual value (FRV) could not be obtained either. In summary, the criteria continuous concentration (CCC) of ferric iron was 9.0 μg/L, and the CMC and CCC derived using the TPR method were 111 μg/L and 9.0 μg/L, respectively.

#### 3.2.3. Results of the SSD Method

**Figure 1:**
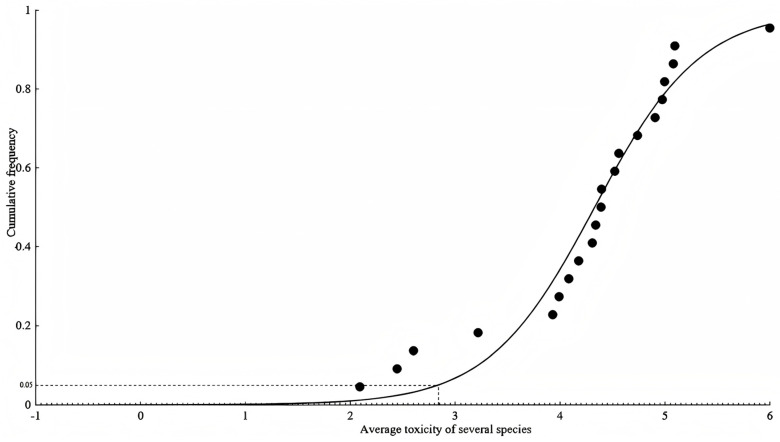
Logistic Steele model fitting based on species-averaged toxicity data using the SSD method.

According to the derivation steps of the SSD method, the SSD model and curve were fitted to the obtained acute water quality criteria of ferric iron. After comparison, the logistic model, with the best goodness-of-fit, was selected as the final derivation model for the acute water quality criteria, as shown in Figure 1 and Table 2. The R^2^ of the fitted curve was 0.96, and the model derived a value of *HC*_5_ of 689 μg/L. With the assessment factor taken to be two, the acute water quality criterium of Fe^3+^ was deduced to be 345 μg/L. Since the amount of chronic toxicity data is insufficient to be used for model fitting, the chronic water quality of Fe^3+^ was derived by dividing *HC*_5_ by the acute-to-chronic ratio (*ACR*), yielding 27.9 μg/L.

#### 3.2.4. Comparison of the WQCs from Three Methods

In this study, three methods were used to derive the WQC of Fe^3+^, and there were some differences among the WQCs obtained from each. Across the three methods, the WQC result from the assessment factor method (AF) was a single value that was relatively low. Using this value might cause “overprotection” for surface aquatic organisms. A single value is also controversial because of the lack of theoretical support from statistics and the high level of uncertainty. Therefore, the WQC derived from the assessment factor method (AF) lacks feasibility. The WQCs derived using the other two methods also have some differences and need to be further analyzed and compared.

Although both methods (TPR and SSD) use the toxicity data of all species, the CMC and LWQC calculated using the TPR method only depend on four generic toxicity values of the most sensitive genera, which means the WQC calculated from the TPR method cannot fully reflect the distribution pattern of all species. In contrast, the species sensitivity distribution (SSD) method fits the model using the toxicity data of all species, and the calculation results are more statistically significant. Based on the SSD, the SWQC and LWQC of Fe^3+^ were 345 μg/L and 27.9 μg/L, respectively.

### 3.3. Ecological Risk Assessment (ERA) of Fe^3+^ in the Regional Surface Water of China

Based on the derived LWQC and SWQC, this study aimed to conduct an ecological risk assessment (ERA) of Fe^3+^ in surface water across provinces in China. The collected Fe^3+^ exposure concentrations in surface water are shown in Table S1 of the Supplementary Materials, covering 30 provinces (there is a total of 34 provincial administrative regions in China; data from Hong Kong, Macao, Inner Mongolia, and Taiwan were not included). The exposure concentration data included the maximum value, minimum value, mean value, and standard deviation (SD). The *Q_R_s* of the mean value within the standard deviation range of each data point were calculated according to the risk quotient method, and the derived risk quotient data were analyzed and evaluated, as shown in Table 3.

According to Table 3, some risk quotients could not be calculated due to the exclusion of four data points resulting in the absence of a mean value; a total of 64 risk quotients were obtained, 31 of which were range data points regarding the standard deviation (SD). Each of the derived risk quotients included a chronic risk quotient from a comparison with the LWQC and an acute risk quotient from the SWQC, both deduced below. The range of acute *Q_R_s* was from 0.0090 to 6.2, while chronic *Q_R_s* ranged from 0.11 to 77.

For acute risk quotients, the mean value of *Q_R_s* for 64 data points was 0.923, proving that the average Fe^3+^ concentration was qualified in the surface water of China. Specifically, the *SQ_R_s* of 19 of 64 data points (30%) exceeded 1, indicating that these surface water systems have excessive levels of Fe^3+^, which can cause adverse impacts on aquatic organisms and human health in the short term. Water quality management is urgently needed in these areas of concern. In addition, the acute risk quotient data comprised 31 data points (48%) and 14 data points (22%), covering the ranges of 0.1–1 and 0.01–0.1, respectively. This indicates that the concentration of Fe^3+^ in these areas meets the requirements of the LWQC but also needs attention. For chronic risk quotients, the average *LQ_R_s* was 11, which is far above the standard. In total, the values of 53 out of the 64 data points (83%) were greater than 1, which is much larger than the relative number of acute risk quotients. This indicates that the toxic effects caused by excessive Fe^3+^ concentration in surface water systems have an obvious time-cumulative effect and that aquatic organisms will exhibit a significant response under long-term impact in most surface water systems in China. Overall, the total concentration of Fe^3+^ and the derived WQC for surface water systems in China is severe; in nearly 30% of the surface water systems, this could cause toxic effects in the short term, while in over 80% of the surface water systems, it would cause harm in the long term.

### 3.4. Obvious Spatial Differences Existed in Fe^3+^ Content in China

In consideration of China’s nine major water systems’ general geographical classification, 68 pieces of data from 30 provinces were divided across eight basins (provinces from the Haihe River Basin and Huaihe River Basin had a high degree of overlap, so they were combined). The means of the acute and chronic risk quotients within each basin were calculated and compared; as shown in Table 4, the acute and chronic *Q_R_s* exhibit highly positive correlations. The surface river from South China (Yangtze River Basin, Southeast Basin, and Pearl River Basin) and the continental area (Continental Basin) have an acute *Q_R_s* below or close to 1 and a chronic *Q_R_s* between 6 and 13, indicating relatively high water quality. In contrast, surface waters from North China (Yellow River Basin, Huaihe River Basin, and Songhua and Liaohe River Basin) show severe problems in water quality, with an average acute *Q_R_s* higher than 2 and chronic *Q_R_s* above 25. Differences in annual precipitation, river velocity, and agriculture and industry types between the north and south may cause the spatial contrast in Fe^3+^.

In addition, Table 5 shows the *Q_R_s* of different reaches in the Yangtze River. Notably, in the same river, lower reaches have an obviously higher toxic effect than upper and middle reaches, demonstrating that anthropogenic discharge could be highly blamed for Fe^3+^ pollution and the WQC.

## 4. Discussion

### 4.1. Comparison and Contrast of the WQC and International Standard Limits

Many countries and non-government environmental organizations (NGEOs) at home and abroad have formulated corresponding standards for the water environment, stipulating the standard limit of iron (Fe) concentration in water bodies. The main stipulated limits are shown in Table 6. The US EPA’s current standard, the “National Recommended Water Quality Criteria”, stipulates the standard limit of aquatic pollutants, involving aquatic organisms’ thresholds, human health standards, and sensory effects. However, for the iron benchmark, only the iron benchmark continuous concentration value (CCC) for the protection of freshwater organisms was put forward as a clear provision. The specific limit value reference QUALITY CRITERIA FOR WATER (Gold Book) was promulgated in 1986. In addition, in the “QUALITY CRITERIA FOR WATER” issued by the U.S. Environmental Protection Agency (EPA) in 1976, the limits of iron in the aquatic environment were stipulated based on the protection of human health and aquatic organisms. In comparing the “GOLD BOOK”, revised in 1986, with the “GOLD BOOK” of 1976, no changes were made to the standard limits for iron. In the WHO’s drinking water standard, “Guidelines for Drinking-water Quality”, the limits for contaminant concentrations are derived from the tolerable daily intake of substances by humans, but the standard does not have a limit value for iron concentrations because the level of iron concentration in drinking water is not considered to have an impact on human health. The European Union’s Drinking Water Quality Directive (Drinking Water Directive) set iron concentration limits for the purpose of protecting human health. Canada, Germany, and France do not have limits for iron concentrations in surface water standards either.

Similarly, China’s current Environmental Quality Standards for Surface Water, implemented in 2002, has been an essential tool for evaluating and assessing the environmental quality of surface water in China, playing an important role in China’s water environment management system. The Hygienic Standard for Drinking Water for Life was implemented in 2007; the water quality standards of many countries or organizations in the world were referenced and revised, while also considering the actual situation in China. In comparison with the standards of other countries, China’s Hygienic Standard for Drinking Water for Domestic Use takes into account the differences between different water sources and meets the requirements of using water from different sources in various regions. However, as compared above, the iron concentration standard limit still needs strict supervision in China. China is still in the stage of continuously improving drinking water safety [67].

### 4.2. Work Limitations and Future Prospects

Although the reliability screening method of toxicity data and the criteria derivation method have been listed in guideline documents and existing studies at home and abroad, biological toxicity data may vary due to different screening principles, subsequently affecting the WQC results. Organisms in different regions may also have differences in drug tolerance. In the future, relevant species will be selected for experimentation to further verify the derived WQC.

Secondly, since most of the dissolved iron in freshwater bodies exists in the form of ferric iron, this study used exposure concentrations in the surface water of total dissolved iron instead of ferric iron, for which the method remains uncertain. There is little research on ferric iron or iron in various water systems in China; much of the literature does not contain the average iron exposure concentration, meaning that reliable analysis cannot be conducted. In various studies, the estimation of ferric iron concentration in freshwater may have been affected by water quality parameters such as pH, dissolved organic matter, and temperature at sampling points, and the current study was unable to quantitatively correct the effects of these factors, which may have resulted in erroneous results in the spatial discussion. At the same time, data on ferric concentration in surface water in the Huaihe River basin, Taiwan Province, and Inner Mongolia should be further collected to comprehensively evaluate the surface water quality.

In addition, the surface water environmental exposure data of iron have a time span of 15 years, which is time-sensitive, and the temporal fate and transfer of ferric iron cannot be analyzed. Future studies should combine environmental monitoring and hydrological monitoring toxicity risk assessment to comprehensively promote the development of surface water system studies.

### 4.3. Contribution to Science

Despite significant progress in deriving water quality benchmarks and conducting ERAs for various heavy metals, such as antimony (Sb) and cadmium (Cd), the collection of toxicity data of Fe^3+^ in aquatic environments remains limited. Moreover, studies have shown that in regions like Hunan and Guizhou in China, low iron concentrations in acid mine drainage-affected water bodies are associated with high mortality rates in aquatic organisms, which highlights the complexity of iron’s ecological impacts and the urgent necessity of deriving biological toxicity benchmarks.

This study systematically collected, screened, and organized aquatic toxicity data from ECOTOX, CNKI, and Web of Science (WOS). A total of 896 studies were collected from WOS with the following keyword searches: TS = iron AND toxicity AND water AND (biological effect OR risk assessment OR benchmarks OR criteria). In addition, 51 Chinese articles from CNKI were obtained. Among them, 47 data points met the requirements of benchmark derivation, in which screening elements included test species, endpoints, exposure time, and experimental methods. Thus, a foundational database was created for Fe^3+^ toxicity benchmarking. The application of the AF, TPR, and SSD methodologies and the derived LWQC and SWQC from the logistic model using the SSD method could serve as a reference for subsequent research. Moreover, this study contributed to ERAs by aggregating a comprehensive, credible dataset of Chinese Fe exposure concentrations, sourced from 49 articles and covering 30 provinces. With an innovated dual-criteria *Q_R_s* approach based on the newly derived LWQC and SWQC, a more precise identification of both acute and chronic Fe risks could be conducted, which is often overlooked in studies employing conventional single-threshold methods. The spatial distribution analysis of iron *Q_R_s* informed the development of targeted regional management and remediation strategies, supporting constructive water quality management and policy decisions.

## 5. Conclusions

Based on the research gap in the derivation of benchmarks for Fe^3+^ in both Chinese and international studies, this study performed a comprehensive assessment of Fe^3+^ toxicity by compiling and screening 47 acute and chronic toxicity data points from 22 species, sourced from multiple literature sources in the ECOTOX, the China National Knowledge Infrastructure, and WOS. Utilizing established methodologies—the AF, TPR, and SSD methods—we derived the LWQC and SWQC for Fe^3+^. Notably, the SSD method, employing logistic fitting curves, was identified as the most appropriate for estimating the acute hazardous concentration for 5% of species (*HC*_5_), yielding a value of 689 μg/L. The derived SWQC was calculated to be 345 μg/L, reflecting a robust short-term guideline for Fe^3+^. In the absence of sufficient chronic toxicity data, the LWQC was estimated by adjusting the acute HC_5_ with an acute-to-chronic ratio (*ACR*), yielding an LWQC of 27.9 μg/L. However, there was a limitation in that the quoted Fe toxicity data included innoxious colloidal FeOOH, resulting in relevantly higher benchmark results.

Subsequent ERA conducted on 68 Fe^3+^ exposure data points across 30 provinces and eight river basins of China demonstrated that approximately 30% of acute risk quotients (*SQ_R_s*) and 83% of *LQ_R_s* exceeded 1, indicating substantial ecological concerns. High-risk values were particularly prevalent in the Yellow River Basin, Huaihe River Basin, and the Songhua and Liaohe River Basins, with the lower reaches exhibiting higher *Q_R_s* than the upper and middle reaches, thus substantiating that anthropogenic discharge is a credible contributor for Fe^3+^. These findings underscore the urgent necessity for region-specific management strategies to address Fe^3+^ contamination and the critical importance of developing and applying accurate benchmarks for effective water quality management.

This study represents a critical step toward filling the existing gap regarding Fe^3+^ toxicity and its environmental impacts, particularly in China. The benchmarks derived from this research can provide a foundational dataset for future regulatory standards and the remediation of iron contamination in surface waters. Moreover, it highlights the importance of further toxicity data collection, particularly chronic exposure studies, to refine water quality criteria and support informed decision making in water quality management and ecosystem protection.

## Figures and Tables

**Table 2:** *LHC*_5_, assessment factor, and LWQC.

*LHC*_5_ (μg/L)	*AF*	LWQC (μg/L)
690 μg/L	2	27.9 μg/L

**Table 3:** Analytical results of Fe^3+^ acute and chronic risk quotients (*Q_R_s*) of exposure concentration in China. The *Q_R_* index equals the reported measured environmental concentration (MEC) divided by our recommended interim predicted no-effect concentration (PNEC); the MEC refers to the selected Fe exposure concentration; and the PNEC was derived using the SWQC (345 μg/L) and LWQC (27.9 μg/L). ND indicates not detected. Risk class: low is *Q_R_* < 0.1, medium is 0.1 < *Q_R_* < 1, high is *Q_R_* > 1; the proportion of each risk class is shown in parentheses.

Risk Class	Low	Medium	High	Mean
*SQ_R_* *s*	14 (22%)	31 (48%)	19 (30%)	0.92
*LQ_R_* *s*	0	11 (17%)	53 (83%)	11

**Table 4:** Proportions of Fe^3+^ acute and chronic risk quotients of exposure concentration in China.

	Effective Data for the Risk Quotient Method	LOW *SQ_R_s* and Medium *LQ_R_s*	*SQ_R_s* and High *LQ_R_s*	*SQ_R_s* and High *LQ_R_s*	*SQ_R_s* and High *LQ_R_s*
Data volume	64	11	3	31	19
Proportion (%)	100	17	55	48	30

**Table 5:** Analysis of Fe^3+^ risk quotient in basins.

Basin (Reaches)	Province (Amount)	Data Amount	Mean of the *SQ_R_s*	Mean of the *LQ_R_s*
Yangtze River Basin, (middle and upper reaches)	Hunan, Hubei, Jiangxi (3)	10	0.58	6.1
Yangtze River Basin, (lower reaches)	Anhui, Jiangsu, Shanghai (3)	8	1.1	13
Southwest Basin	Tibet, Sichuan, Chongqing, Guizhou, Yunnan (5)	18	0.56	7.0
Pearl River Basin	Guangdong, Guangxi, Hainan (3)	7	0.92	11
Southeast Basin	Fujian, Zhejiang (2)	3	0.78	9.7
Yellow River Basin and Huaihe River Basin	Beijing, Tianjin, Hebei, Shanxi (4)	8	2.2	27
Songhua and Liaohe River Basin	Heilongjiang, Jilin, Liaoning (3)	2	2.3	28
Continental Basin	Xinjiang, Ningxia, Gansu (3)	5	0.65	8.0

**Table 6:** Standard limits for iron according to different countries and organizations.

Countries or Organizations	Standard	Category	Limit (μg/L)
United States	National Recommended Water [59]	CCC	1000
	Quality Criteria for Water 1976 [60]	domestic water	300
		aquatic organisms	1000
	Quality Criteria for Water 1986 [61]	domestic water	300
		aquatic organisms	1000
WHO	Guidelines for Drinking-water Quality [62]	-	-
EU	Drinking Water Directive [63]		200
Canada	Drinking Water Standards [64]	-	300
Germany	Drinking Water Standards [65]	-	200
France	Drinking Water Standards [66]	-	200
China	Surface Water Quality Standard (GB 3838-2002) [6]	-	300
	Drinking Water Standard (GB 5749-2006) [7]	-	300

## Data Availability

The original data presented in the study are openly available in the [Ecotoxicology Database (ECOTOX)] or the [China National Knowledge Infrastructure] or the [Web of Science (WOS) Database].

## Supplementary Materials

The following supporting information can be downloaded at https://www.mdpi.com/article/10.3390/toxics13060475/s1, Table S1: Ecological risk assessment quantified using the risk quotient approach based on Fe exposure concentrations in China. References [15,47,49,68,69,70,71,72,73,74,75,76,77,78,79,80,81,82,83,84,85,86,87,88,89,90,91,92,93,94,95,96,97,98,99,100,101,102,103,104,105,106,107,108,109,110,111,112,113] are cited in the supplementary materials.
