# Supplementary material for: Derivation of a Freshwater Quality Benchmark and an Ecological Risk Assessment of Ferric Iron in China
Source: Toxics. 2025 Jun 4;13(6):475. doi: 10.3390/toxics13060475 (PMC12197261; doi:10.3390/toxics13060475)
Supplement: Supplementary file 1 [file toxics-13-00475-s001.zip › toxics-3603898-supplementary.pdf]

The data from Table S1 was collected from Chinese Core Journals indexed in the China National Knowledge Infrastructure Database (CNKI) and WanFang Data Knowledge Service Platform, ensuring the credibility and reliability. Our efforts show in both comprehensive dataset integration and the dual-criteria RQ (generated from both acute and chronic benchmarks) approach. The collected Fe exposure concentration dataset covered a wide geographic range, extended time span, and diverse sources. Besides, the newly derived short-term (LWQC) and long-term (SWQC) water quality criteria in the main text were used in dual-benchmark calculation of both chronic and acute risk quotients (CRQs & ARQs), allowing for a more targeted assessment of ecological risks. For instance, in Bozhou, Anhui, the chronic RQ remained low (0.089), while the acute RQ revealed significant short-term risk (1.1), uncovering ecological risks more precisely about which conventional single-criterion RQ approaches overlooked.

Table S1. Ecological risk assessment quantified using a risk quotient (RQ) approach based on Fe exposure concentration in China.

Province	Location[a]	Mean Fe exposure concentration (ug/L)	Risk quotient[b]		Standard deviation (SD, ug/L)	Concentration (MEC, ug/L)[c]	Data Sources	Acute risk quotient[d]	Chronic risk quotient[d]	Actue risk class[e]	Chronic risk class[e]
			Acute	Chronic							
Shanxi	Fenhe River upper reaches	426.45	1.238	15.285	631.73	ND	[1]	1.238	15.285	High	High
	Middle reaches	2139.09	6.208	76.67	1590.64	548.45~3729.73		1.592~10.824	19.658~133.682	High	High
	Lower reaches	2088.89	6.062	74.871	1227.89	861.00~3316.78		2.449~9.626	30.860~118.881	High	High
	Tributaries	556.25	1.614	19.937	321.6	234.65~877.85		0.681~2.548	8.41~ 31.464	High	High
Shaanxi	Weihe River	382.41	1.11	13.706	164.68	217.73~547.09	[2]	0.632~1.588	7.804~ 19.609	High	High

Shaanxi	Chuhe River Wet Season	135.93	0.394	4.872	124.93	11~260.86	[3]	0.032~ 0.757	0.394~~9.350	Medium	High
	Chuhe River Dry Season	229.57	0.666	8.228	51.64	177.93~281.21		0.516~ 0.816	6.377~ 10.079	Medium	High
Jiangsu	Yangtze River, Nanjing	239.8	0.696	8.595	56.1	183.7~295.9	[4]	0.533~ 0.859	6.584~ 10.606	Medium	High
Jiangsu	Changzhou	130.03	0.377	4.661	16.69	113.34~146.72	[5]	0.329~ 0.426	4.062~ 5.259	Medium	High
Jiangsu	Zhenjiang	3.03	0.009	0.109	9.83	ND	[6]	0.009	0.109	Low	Medium
Anhui	Bozhou	30.1	0.087	1.079	23.96	6.14~54.06	[7]	0.018~ 0.157	0.220~ 1.938	Low	High
Anhui	Wabu Lake	257.96	0.749	9.246	ND	ND	[8]	0.749	9.246	Medium	High
Anhui	Huai River, Fuyang	1342	3.895	48.1	ND	ND	[9]	3.895	48.1	High	High
Hunan	Huayuan County	152	0.441	5.448	135	17~287	[10]	0.049~0.833	0.609~10.287	Medium	High
Liaoning	Upper Reaches of Hunhe River	ND	ND	ND	ND	ND	[11]	-	-	-	-
Henan	Lunhun Reservoir	30.05	0.087	1.007	14.45	15.6~44.5	[12]	0.045~ 0.129	0.559~ 1.595	Low	High
Hubei	Danjiangkou	19.14	0.056	0.686	31.75	ND	[13]	0.056	0.686	Low	Medium
Hubei	Lushui Reservoir	6.219	0.018	0.223	ND	ND	[14]	0.018	0.223	Low	Medium

Yunnan	Qiantun Reservoir	357.7	1.038	12.821	ND	ND	[15]	1.038	12.821	High	High
	Qingmeijian Reservoir	301.5	0.875	10.806	ND	ND		0.875	10.806	Medium	High
	Wenbihai Reservoir	5.7	0.017	0.204	ND	ND		0.017	0.204	Low	Medium
	Erhai Lake	53.5	0.155	1.918	ND	ND		0.155	1.918	Medium	High
Yunnan	Xiaohuangni River	810.66	2.353	29.056	1323.39	ND	[16]	2.353	29.056	High	High
Chongqing	Xiaonanhai Reservoir, Qianjiang	6	0.017	0.215	ND	ND	[17]	0.017	0.215	Low	Medium
Chongqing	Liangtan River	367.2	1.066	13.16	170.84	196.36~538.04	[18]	0.570~ 1.561	7.038~ 19.285	High	High
Guangxi	Jiuzhou River	436	1.265	15.627	219	217~655	[19]	0.630~ 1.901	7.778~ 23.477	High	High
Guangxi	Liujiang River, Upper Reaches	150.85	0.438	5.407	10.05	140.8~160.9	[20]	0.409~ 0.467	5.047~ 5.767	Medium	High
	Liujiang River, Middle Reaches	357.2	1.037	12.803	38.05	319.15~395.25		0.926~ 1.147	11.439~14.167	High	High
	Liujiang River, Lower Reaches	122.91	0.357	4.405	11.44	111.47~134.35		0.324~ 0.390	3.995~ 4.815	Medium	High
Tibet	Yangzhuoyongcuo	10.41	0.03	0.373	ND	ND	[21]	0.030	0.373	Low	Medium

Tibet	Niyang River, Normal Season	305.5	0.887	10.95	ND	ND	[22]	0.887	10.95	Medium	High
	Niyang River, Dry Season	617.4	1.792	22.129	ND	ND		1.792	22.129	High	High
	Niyang River, Wet Season	97.8	0.284	3.505	ND	ND		0.284	3.505	Medium	High
Tibet	Lhasa River	11.829	0.034	0.424	ND	ND	[23]	0.034	0.424	Low	Medium
Jiangxi	Poyang Lake	33.69	0.098	1.208	2.47	31.22~36.16	[24]	0.091~ 0.105	1.119~ 1.296	Low	High
Jiangxi	Fuhe River, Nanchang, Normal Season	372.27	1.808	13.343	284.02	88.25~656.29	[25]	0.256~ 1.905	3.163~ 23.523	High	High
	Fuhe River, Nanchang, Dry Season	416.85	1.21	14.941	771.99	ND		1.21	14.941	High	High
	Fuhe River, Nanchang, Wet Season	305.09	0.885	10.935	280.07	25.02~585.16		0.073~ 1.698	0.897~ 20.973	Medium	High
Jiangxi	Pooyang Lake	80	0.232	2.867	118.6	ND	[26]	0.232	2.867	Medium	High
Jiangxi	Xin River	188.03	0.546	6.739	153.43	34.6~341.46	[27]	0.100~ 0.991	1.240~ 12.239	Medium	High
Jiangxi	Ping River	128.2	0.372	4.594	ND	ND	[28]	0.372	4.594	Medium	High

Ningxia	Qingshuihe River, Normal Season	225	0.653	8.065	83.25	141.75~308.25	[29]	0.411~ 0.895	5.081~ 11.048	Medium	High
	Qingshuihe River, Dry Season	366	1.062	13.118	115.66	250.34~481.66		0.727~ 1.398	8.973 ~17.264	High	High
	Qingshuihe River, Wet Season	276	0.801	9.892	196.51	79.49~472.51		0.231~ 1.371	2.849~ 16.936	Medium	High
Shanghai	Huangpu River	910	2.641	32.616	ND	ND	[30]	2.641	32.616	High	High
	Dianshan Lake	49	0.142	1.756	ND	ND		0.142	1.756	Medium	High
Tianjin	Hehai River	57.12	0.166	2.047	ND	ND	[31]	0.166	2.047	Medium	High
Hainan	Wanquan River	37	0.107	1.326	11	26~48	[32]	0.075~ 0.139	0.932~ 1.720	Medium	High
Guangdong	Zhuhai	3.98	0.012	0.143	ND	ND	[33]	0.012	0.143	Low	Medium
Guangdong	Dongjiang River	ND	ND	ND	ND	ND	[34]	-	-	-	-
Guangdong	Pearl River Delta	1100	3.192	39.427	70	1030~1170	[35]	2.989~ 3.396	36.918~41.935	High	High
Guizhou	Guiyang, Baihua Lake	45.32	0.132	1.624	ND	ND	[36]	0.132	1.624	Medium	High
Guizhou	Huaxi Reservoir	51.46	0.149	1.844	ND	ND	[36]	0.149	1.844	Medium	High

Henan and Hebei	South-to-north Water-diversion Canal	199.27	0.578	7.142	136.91	62.36~336.18	[37]	0.181~ 0.976	2.235~ 12.049	Medium	High
Sichuan	Chengdu, Wet Season	49.711	0.144	1.782	ND	ND	[38]	0.144	1.782	Medium	High
	Chengdu, Dry Season	55.308	0.161	1.982	ND	ND		0.161	1.982	Medium	High
Sichuan	Guangyuan	116.6	0.367	4.538	ND	ND	[39]	0.367	4.538	Medium	High
Sichuan	Xingjing County	222.42	0.646	7.972	ND	ND	[40]	0.646	7.972	Medium	High
Fujian	Min River	ND	ND	ND	ND	ND	[41]	-	-	-	-
Heilongjiang	Xianhe Lake	6.42	0.019	0.23	ND	ND	[42]	0.019	0.23	Low	Medium
Jilin	Siping	1550	4.498	55.555	9	1541~1559	[43]	4.472~ 4.524	55.233~55.878	High	High
Zhejiang	Ningbo, Wet Season	580	1.683	20.789	97	483~677	[44]	1.402~ 1.965	17.312~24.265	High	High
	Ningbo, Dry Season	210	0.609	7.567	77	133~287		0.386~ 0.833	4.767~ 10.287	Medium	High
Zhejiang	Xiangshan Bay Watershed	18.67	0.054	0.669	7.2	11.47~25.87	[45]	0.033~ 0.075	0.411~ 0.927	Low	Medium
Gansu	Maqu County	72.2	0.21	2.588	78.9	ND	[46]	0.21	2.588	Medium	High
Xinjiang	Gongnaisi River	181.833	0.528	6.517	ND	ND	[47]	0.528	6.517	Medium	High

Beijing	Miyun, Chaobai River	9.29	0.027	0.333	5.11	4.18~14.4	[48]	0.012~ 0.042	0.150~ 0.516	Low	Medium
Shandong	Nansi Lake	ND	ND	ND	ND	ND	[49]	-	-	-	-

[a] Fe Concentration data from different reaches or seasons of the same location was distinguished.

[b] RQ was calculated by the reported measured environmental concentration (MEC, column "Mean Fe exposure concentration") divided by the recommended interim predicted no effect concentration (PNEC, derived SWQC (345 μg/L) and LWQC (27.9μg/L))

[c] Concentration was represented as the mean value ± standard deviation (mean ± SD); ND indicates not detected

[d] Acute and chronic risk quotient were calculated by the reported measured environmental concentration (MEC, column "Concentration") divided by the recommended interim predicted no effect concentration (PNEC, derived SWQC (345 μg/L) and LWQC (27.9μg/L))

[e] Risk class classification: Low is RQ<0.1, Medium is 0.1<RQ<1, High is RQ>1.

1. Zhao, C.H., et al., *Distribution characteristics and health risk assessment of metal elements in surface water of Fenhe River Basin.* Environmental Chemistry, 2024. **43**(02): p. 549-560.

2. Ren, L.J., et al., *Pollution characteristics and health risk assessment of heavy metals in surface water in Guanzhong Section of the Weihe River Basin.* Ecology and Environmental Sciences, 2022. **31**(01): p. 131-141.

3. Guo, Y.K., et al., *Spatial and temporal distribution characteristics and health risk assessment of heavy metals in the Chu River basin.* . Environmental Engineering, 2023. **41**(01): p. 112-119.

4. Wu, B., et al., *Preliminary risk assessment of trace metal pollution in surface water from Yangtze River in Nanjing Section, China.* Bull Environ Contam Toxicol, 2009. **82**(4): p. 405-9.

5. Chen, Y.F., et al., *Pollution assessment and source analysis of heavy metals in the Wunan River, Changzhou.* Acta Scientiae Circumstantiae, 2024. **44**(03): p. 157-166.

6. Wang, C.X., J. Huang, and Z.W. Yang, *Study on dynamic change feature of heavy metals in Zhenjiang Section of Yangtze River and their effects on health risks of residents.* . Environmental Protection and Technology, 2022. **28**(06): p. 22-27.

7. Si, W., et al., *Distribution characteristics and risk assessment of dissolved metal (loid)s in typical river basin of Hozhou City.* . Acta Scientiae Circumstantiae, 2024. **44**(2).

8. Li, G.L., et al., *Enrichment characteristics, risk evaluation, and source apportionment of heavy metals in Wabu Lake of Yangtze River Water Diversion Project.* Environmental Science, 2024. **45**(12): p. 7111-7122.

9. Shu, Z.C. and C. Cheng, *Distribution characteristics and pollution evaluation of heavy metals in Fuyang Section of Huaihe River.* Journal of Green Science and Technology, , 2023. **25**(10): p. 157-160.

10. Liu, Y.B., et al., *Assessment of heavy metal pollution and health risks in a main river in Huayuan County,Xiangxi,Hunan Province.* Chinese Journal of Applied and Environmental Biology, 2018. **24**(3): p. 0602-0608.

11. Ma, Y.Q., et al., *Temporal-spatial distribution and pollution assessment of heavy metals in the Upper Reaches of Hunhe River (Qingyang Section), northeast China.* . Environmental Science, 2014. **35**(1): p. 108-116.

12. Yu, C.C., et al., *Distribution characteristics and health risks assessment of metals in drinking water sources from the Luhun Reservior.* . Environmental Science, 2018. **39**(01): p. 89-98.

13. Li, S.Y., et al., *Dissolved trace elements and heavy metals in the Danjiangkou Reservoir, China.* Environmental Geology, 2008. **55**(5): p. 977-983.

14. Mi, W.J., et al., *Contents and risk assessment of heavy metals in water environment of Lushui Reservoir.* Environmental Science & Technology, 2023. **46**(S2): p. 9-16.

15. Liu, Z.T., C.H. Li, and G.Y. Zhang, *Application of principal component analysis to the distributions of heavy metals in the water of lakes and reservoirs in Yunnan Province.* Research of Environmental Sciences, 2010. **23**(4): p. 459-466.

16. Tu, C.L., et al., *Distribution and health risk assessment of heavy metals in Xiaohuangni River Basin on Yunnan-Guizhou Plateau.* Environmental Chemistry, 2023. **42**(12): p. 4238-4252.

17. Zhang, Y.J., et al., *Health risk assessment of metals form drinking water sources in Chongqing Ecological Protection Area.* The Administrition and Technique of Environrmental Monitor, 2017. **29**(3).

18. He, S.J., et al., *Health Risk Assessment and Source Analysis of Heavy Metal Pollution in the Liangtan River Basin, Chongqing City.* Journal of Hydroecology. **44**(4).

19. He, Y., X. Hong, and X.Y. Bi, *Characteristic and sources of heavy metal pollution in water environment of Jiuzhou River basin.* Environmental Chemistry, 2021. **40**(1): p. 240-253.

20. Zhang, Q.H., et al., *Heavy metal pollution of the drinking water sources in the Liujiang River Basin, and related health risk assessments.* Environmental Science, 2018. **39**(4).

21. Zhang, M., et al., *Spatial distribution characteristics and health risk assessment of heavy metals in Yamdrok Lake, Tibet.* Water Resources & Hydropower of Northeast China, 2024. **3**: p. 23-28.

22. Lü Linli, L.Z., Huang Yi, Cui *Chongyu. Distribution characteristics and risk assessment of heavy metals in Niyang River, Tibet. .* Transactions of the Chinese Society of Agricultural Engineering (Transactions of the CSAE), 2019. **35**(9): p. 193 – 199.

23. Qin, H.H., et al., *Distribution characteristics and pollution risk assessment of heavy metals in Lhasa River Basin.* Nonferrous Metals (Extractive Metallurgy), 2020. **10**: p. 79-86.

24. Wang, Z.G., et al., *Distribution of heavy metal and human health risk assessment of water bodies in the Basin of Poyang Lake into the Yangtze River.* Resources and Environmental in the Yangtze Basin 2023. **32**(6).

25. Chen, J.H., *Study on pollution characteristics and health risk of heavy metals in Nanchang section of the Fuhe River*, in *Hydraulic Engineering*. 2021, East China University of Technology.

26. Zhao, Q.L., et al., *Pollution characteristics and human health risk assessment of heavy metals in water bodies around Poyang Lake.* Nonferrous Metals Engineering, 2024. **14**(8).

27. Ge, Q., B. Gao, and C.Y. Lin, *Sources of heavy metals and relaled heallh risk of surfke watef in xinjiang River Basin.* Science Technology and Engineering, 2023. **23**(12).

28. Zhang, Y.H., et al., *Pollution characteristics and health risk assessment of heavy metals in surface water of Pingshui River.* Nonferrous Metals (Extractive Metallurgy), 2021. **7**: p. 116-125.

29. Zhao, Z.F., et al., *Distribution characteristics and health risk assessment of five heavy metals in the w of Qingshuihe River Basin.* The Administrition and Technique of Environrmental Monitor, 2021. **33**(3): p. 35-40.

30. Pu, Y.L., *Heavy metal contamination pollution assessment and source analysis of natural water sediments in Shanghai*, in *Environmental Science*. 2018, Shanghai Normal University Master of Philosophy.

31. Shi, X.S., *The time and space distribution and geochemical characteristics of heavy metals in Haihe river trunk stream water*, in *Environmental Science*. 2014, Tianjin Normal University.

32. Xin, C.L., et al., *Distributions and pollution status of heavy metals in the suspended particles of the Esyuaries and Coastal Area of Eastern Hainan* Environmental Science, 2013. **34**(4): p. 1315-1323.

33. Wang, Q., Z.Y. Mai, and Y. Zhao, *Characteristics of heavy metal content and health risk Assessment in centralized drinking water sources in western Zhuhai.* Shandong Chemical Industry, 2024. **53**: p. 274-278.

34. Wang, R.S., et al., *Health risk assessment of heavy metals in typical township water sources in Dongjiang River Basin.* Environmental Science, 2012. **33**(9): p. 3083-3088.

35. Xie, W.P., et al., *Evaluation on heavy metal contents in water and fishes collected from the waterway in the Pearl River Delta, South China.* Journal of Agro-Environment Science, 2010. **29**(10): p. 1917-1923.

36. Wang, H.H., et al., *Pollution characteristics and health risk assessment of heavy metals in drinking water source of Guiyang.* Ecology and Environmental Sciences, 2022. **31**(10): p. 2039-2047.

37. Guo, Y.Y., et al., *Contents and risk assessment of heavy metals in surface water in the main canal of middle route of the South-to-North Water Diversion Project.* Acta Ecologica Sinica, 2022. **46**(7): p. 995-1006.

38. He, X., Y.J. Zhang, and C.Y. LAi, *Preliminary assessment on health risks of heavy metals in drinking water sources in Chengdu City.* Water Resources Development and Management, 2023. **6**.

39. Zheng, Q., et al., *Preliminary assessment of health risk of heavy metals in urban drinking water source of Guangyuan City from 2012-2014.* Sichuan Environment, 2020. **39**(6): p. 172-178.

40. Zhao, R.Y., et al., *Distribution characterictics of heavy metals and health risk assessment for Yingjing River.* Ecological and Environmental Monitoring of Three Gorges, 2021. **6**(4): p. 24-31.

41. Wu, J.W., et al., *Environmental health risk assessment of heavy metals in drinking water source based on cloud model.* Journal of Fuzhou University (Natural Science Edition), 2014. **42**(2): p. 327-332.

42. Sun, Q.Z., et al., *Heavy metal pollution and its potential ccological risk assessment of Xianhe Lake.* Chinese Agriculture Science Bulletin, 2012. **28**(2): p. 261-266.

43. Liu, F., et al., *Monitoring and health risk assessment of Heavy Metals in River of Spring City.* Shandong Chemical Industry, 2017. **46**: p. 176-178.

44. Xu, M.J., et al., *Temporal-spatial distribution and health risk assessment of heavy metals in the surface water of Ningbo.* Environmental Science, 2023. **44**(3): p. 1407-1415.

45. WAng, L., et al., *Risk assessment and source analysis of heavy metals in the river of a typical bay watershed.* Environmental Science, 2020. **41**(7): p. 3194-3203.

46. Zhang, G.Z., et al., *Water quality assessment and heavy metal health risk evaluation in Northwest Alpine Pastoral Area.* China Water & Wastewater, 2023. **39**(11).

47. Li, M.Y., et al., *Characteristic analysis of trace heavy metal elements of Kunes River.* Water Resources Protection, 2009. **25**(5).

48. Xu, X.Y., X. Pu, and X.L. Liu, *Tempo-spatial variations of dissolved organic carbon and heavy metals in Miyun Section of Chaobai River.* Journal of Ecology and Rural Environment, 2020. **36**(9): p. 1177 – 1184.

49. Ye, D.Y., *Water environment quality status of inlet streams and its response to the spatial pattern of land use in the watershed of Nansihu Lake*, in *Geography*. 2022, Qufu Normal University.
